# Decreased microRNA-224 and its clinical significance in non-small cell lung cancer patients

**DOI:** 10.1186/s13000-014-0198-4

**Published:** 2014-11-19

**Authors:** Dan Zhu, Hui Chen, Xiguang Yang, Weisong Chen, Linying Wang, Jilin Xu, Long Yu

**Affiliations:** Department of Respiratory Medicine, Jinhua Municipal Central Hospital, Jinhua, 321000 P.R. China

**Keywords:** MicroRNA-224, Non–small cell lung cancer, Prognosis, Proliferation, Apoptosis, Invasion

## Abstract

**Background:**

MicroRNA-224 has been proven dysregulated in some human malignancies and correlated with tumor progression. However, its expression and clinical significance in non–small cell lung cancer (NSCLC) is still unclear. Thus, the aim of this study was to explore the effects of miR-224 in NSCLC tumorigenesis and development.

**Methods:**

Using real-time quantitative RT-PCR, we detected miR-224 expression in NSCLC cell lines and primary tumor tissues. The association of miR-224 expression with clinicopathological factors and prognosis was also statistically analyzed. MTT, flow cytometric, Transwell invasion and migration assays, and scratch migration assay were used to test the proliferation, apoptosis, invasion, and migration of NSCLC cells after miR-224 mimics transfection.

**Results:**

MiR-224 expression levels were significantly down-regulated in NSCLC compared to the corresponding noncancerous lung tissues (P <0.001). In addition, decreased miR-224 expression was significantly associated with lymph node metastasis (P = 0.002), advanced TNM stage (P <0.001), and shorter overall survival (P <0.001). Multivariate regression analysis corroborated that down-regulation of miR-224 was an independent unfavourable prognostic factor for patients with NSCLC. Furthermore, transfection of miR-224 mimics in NSCLC A549 cells was able to reduce cell proliferation, invasion, and migration, and promote cell apoptosis.

**Conclusions:**

These findings indicate that miR-224 may act not only as a novel diagnostic and prognostic marker, but also as a potential target for miR-based therapy of NSCLC.

**Virtual Slides:**

The virtual slide(s) for this article can be found here: http://www.diagnosticpathology.diagnomx.eu/vs/13000_2014_198

## Background

Lung cancer is the leading cause of cancer related deaths worldwide [1]. Despite advances in the fields of oncology and surgery, the prognosis of lung cancer has not improved significantly over several decades [2]. Non–small cell lung cancer (NSCLC) is the predominant group of lung cancer. To date, the highly complex molecular mechanisms underlying NSCLC carcinogenesis and progression remain poorly understood, and no appropriate biomarker exists to detect NSCLC at early stages. Therefore, it is necessary to search novel markers for NSCLC, which can accurately identify biological characteristics of tumors, improve therapeutic strategies, and predict clinical outcome.

MicroRNAs (miRNAs) are single-stranded, small noncoding RNAs with 18–25 nucleotides in length [3]. They can negatively regulate gene expression through base-pairing to the 3′ untranslational region (3′UTR) of target messenger RNA (mRNA), resulting in translation inhibition or mRNA degradation [4,5]. Beyond the involvement in diverse biological processes, including cell growth, apoptosis, development, differentiation and endocrine homeostasis [6], emerging evidence strongly suggests that the deregulation or dysfunction of miRNAs contributes to human carcinogenesis and cancer progression [7-9]. miRNAs can function as either oncogenes or tumor suppressors according to the roles of their target genes. In terms of NSCLC, *in vitro* functional assays showed that both miR-31 and miR-196 promote the proliferation, invasion, and migration of cancer cells [10,11]. Clinical analysis demonstrated that decreased miR-375 and increased miRNA-21 expression in NSCLC tissues were associated with advanced clinical stage and poor prognosis [12,13]. Furthermore, Bian et al. reported that upregulation of miR-451 sensitized NSCLC A549 cells to cisplatin [14]. Wang et al. found that knock-down of miRNA-21 promoted the radio-sensitivity of A549 cells [13]. These findings indicate that miRNAs may act not only as diagnostic and prognostic markers, but also as potential therapeutic targets of human NSCLC.

One of the cancer-related miRNAs is miR-224. Aberrant expression of miR-224 in human malignancies has been demonstrated to play various roles in tumorigenesis. The expression level of miRNA-224 was downregulated in oral cancer [15], ovarian cancer [16], prostate cancer [17], malignant giant cell tumor [18], and glioblastoma [19]; while it was upregulated and functioned as an oncogene in hepatocellular carcinoma [20], clear cell renal cell carcinoma [21], pancreatic cancer [22], and cervical cancer [23]. Notably, a previous study by Yanaihara et al. detected decreased miR-224 levels in human lung cancer tissues using miRNA microarray analysis [24]. However, currently, little is known about the links of miR-224 dysregulation to clinicopathological characteristics of NSCLC, and the functional attributes of miR-224 associated with NSCLC progression have not been experimentally established.

In the present study, we examined miR-224 expression in NSCLC tissues and cell lines using real-time PCR. The association of miR-224 levels with clinicopathologic features and prognosis was also analyzed. Furthermore, we investigated the effects of miR-224 on proliferation, apoptosis, invasion and migration of NSCLC cells.

## Methods

### Patients and tissue samples

This study was approved by the Research Ethics Committee of Jinhua Municipal Central Hospital (Jinhua, Zhejiang province, People’s Republic of China). Written informed consent was obtained from all of the patients. All specimens were handled and made anonymous according to the ethical and legal standards.

One hundred and fifteen pairs of primary NSCLC and adjacent noncancerous tissues (>2 cm from the cancer tissue, in the same lobe) were collected at the time of surgery from patients who underwent surgical resection at Jinhua Municipal Central Hospital from January 1, 2007 to December 30, 2009. There were 77 men (67%) and 38 women (33%) with median age of 60 years at the time of diagnosis. The selection criteria were as follows: (1) pathologically confirmed patients with NSCLC; (2) no evidence of distant metastases. Patients were excluded if they had a previous or secondary malignancy, and/or had undergone chemotherapy, radiation therapy or immunotherapy before surgery. All tissues were immediately frozen in liquid nitrogen and stored at −80°C until use. Clinicopathological information was shown in Table 1. Smoking intensity was evaluated according to pack years, which were calculated by multiplying the number of cigarette packs (20 cigarettes per pack) smoked per day by the number of years of smoking. High risk jobs meant occupational exposure to carcinogens such as asbestos and silica dust. Clinical follow-up was available for all patients. Overall survival (OS) was defined as the time from primary surgery to death of the patient or, for living patients, the date of last follow-up.

**Table 1:** **Correlation between miR-224 expression and different clinicopathological features in non–small cell lung cancer**

**Clinicopathological features**	**No. of cases**	**miR-224 expression**		**P**
		**Low (n, %)**	**High (n, %)**	
Age				
<60	58	34 (58.6%)	24 (41.4%)	0.094
≥60	57	24 (42.1%)	33 (57.9%)	
Gender				
Male	77	40 (51.9%)	37 (48.1%)	0.695
Female	38	18 (47.4%)	20 (52.6%)	
Smoking status				
Smoking	68	38 (55.9%)	30 (44.1%)	0.591
No smoking	47	20 (42.6%)	27 (57.4%)	
Smoking intensity (for smokers)				
<30 pack years	38	17 (44.7%)	21 (55.3%)	0.484
≥30 pack years	30	17 (56.7%)	13 (43.3%)	
Occupational exposure				
Yes	32	14 (43.8%)	18 (56.2%)	0.411
No	83	44 (53.0%)	39 (47.0%)	
Histological type				
Squamous cell carcinoma	40	23 (57.5%)	17 (42.5%)	
Adenocarcinoma	61	26 (42.6%)	35 (57.4%)	0.186
Others	14	9 (64.3%)	5 (35.7%)	
Histological grade				
G1 + G2	61	27 (44.3%)	34 (55.7%)	0.192
G3	54	31 (57.4%)	23 (42.6%)	
T classification				
T_1+2_	77	36 (46.8%)	41 (53.2%)	0.323
T_3_	38	22 (57.9%)	16 (42.1%)	
N classification				
Positive	80	48 (60.0%)	32 (40.0%)	0.002
Negative	35	10 (28.6%)	25 (71.4%)	
TNM stage				
I + II	69	25 (36.2%)	44 (63.8%)	<0.001
III	46	33 (71.7%)	13 (28.3%)	

### Cell lines and culture conditions

Four NSCLC cell lines (A549, H460, 95D, and H358) and a normal human bronchial epithelial cell line (16HBE) were purchased from the Institute of Biochemistry and Cell Biology of the Chinese Academy of Sciences (Shanghai, China). Cells were cultured in RPMI 1640 medium (Invitrogen, Gaithersburg, MD, USA) supplemented with 10% fetal bovine serum (10% FBS), 100 U/ml penicillin, and 100 *u*g/ml streptomycin in humidified air at 37°C with 5% CO_2_.

### RNA extraction and quantitative real-time PCR

Total RNA was isolated using TRIzol® reagent (Invitrogen Corp, Carlsbad, CA, USA) according to the manufacturer’s instructions. Reverse transcription reaction was carried out starting from 100 ng of total RNA using the looped primers. Real-time PCR was performed using the standard Taqman MicroRNA assays protocol on ABI7500 real-time PCR detection system with cycling conditions of 95°C for 10 min, followed by 40 cycles of 95°C for 15 s and 60°C for 60 s. U6 small nuclear RNA was used as an internal control. The PCR primers for mature miR-224 or U6 were designed as follows: miR-224 forward, 5′- CACTAGTGGTTCCGTTTAGTAG -3′ and reverse, 5′- TTGTAGTCACTAGGGCACC -3′. U6 forward, 5′- CTCGCTTCGGCAGCACA-3′ and reverse, 5′-AACGCTTCACGAATTTGCGT-3′. The threshold cycle (Ct) was defined as the fractional cycle number at which the fluorescence passed the fixed threshold. Each sample was measured in triplicate, and the relative amount of miR-224 to U6 was calculated using the equation 2^−ΔCt^, where ΔCT = (CT^miR-224^ - CT^U6^).

### Cell transfection

For RNA transfection, the cells were seeded into each well of 24-well plate and incubated overnight, then transfected with either miR-224 mimics (GenePharma, Shanghai, China) or negative control (NC) RNA-oligonucleotides (GenePharma) using Lipofectamine 2000 (Invitrogen, California, USA) in accordance with the manufacturer’s procedure. The transfection efficiency of miR-224 mimics was confirmed by real-time PCR analysis.

### MTT assay

Cells were seeded into 96-well culture plates at a density of 2,000 cells in 200 *u*L/well and incubated at 37°C after transfection. 100 *u*L of MTT solution (0.5 mg/mL; Sigma, USA) was added to each well, and the cells were incubated for another 4 hours. Then the medium was replaced with 150 *u*L of DMSO. Spectrometric absorbance at 490 nm was measured using a microplate reader. Cell proliferation was assessed daily for 4 consecutive days, and the MTT assay was repeated 3 times.

### Detection of apoptosis by flow cytometry

Apoptosis was detected by flow cytometric analysis. Briefly, the cells were washed and resuspended at a concentration of 1 × 10^6^ cells/mL. Then, the cells were stained with Annexin V and propidium iodide (PI), using the Annexin V apoptosis detection kit. After incubation at room temperature in the dark for 15 min, the cell apoptosis was analyzed on a FACSCalibur (Becton, Dickinson and Company, San Jose, CA).

### Transwell migration and invasion assays

The migration and invasion assays were performed using 24-well transwell chambers (8 μm; Corning). For the migration assay, tumor cells were resuspended in serum-free RPMI-1640 medium and 2 × 10^5^ cells were seeded into the upper chambers. 0.5 mL RPMI-1640 containing 10% FBS was added to the bottom chambers. Following a 24 h-incubation, cells on the upper surface of the membrane were scrubbed off, and the migrated cells were fixed with 95% ethanol, stained with 0.1% crystal violet, and counted under a light microscope. The invasion assay protocol was similar to that of the migration assay except that the upper chambers were first covered with 1 mg/mL Matrigel.

### Scratch migration assay

Scratch migration assay was also performed to confirm the influence of miR-224 on NSCLC cell migration. When the cells transfected with miR-224 mimics or NC were grown to confluence, a scratch in the cell monolayer was made with a cell scratch spatula. After the cells were incubated under standard conditions for 24 h, pictures of the scratches were taken by using a digital camera system coupled with a microscope.

### Statistics

Statistical analyses were carried out using SPSS software (version 16.0, SPSS Inc, IL, USA). Data were expressed as mean ± standard deviation (SD). The differences between groups were analyzed using the Student’s t-test, chi-square test or Fisher’s exact test. Patient survival curves were estimated by the Kaplan-Meier method. The joint effect of covariables was examined using the Cox Proportional Hazard Regression Model. All tests were two-tailed, and the significance level was set at P <0.05.

## Results

### Decreased expression of miR-224 in NSCLC tumor samples and cell lines

**Figure 1:**
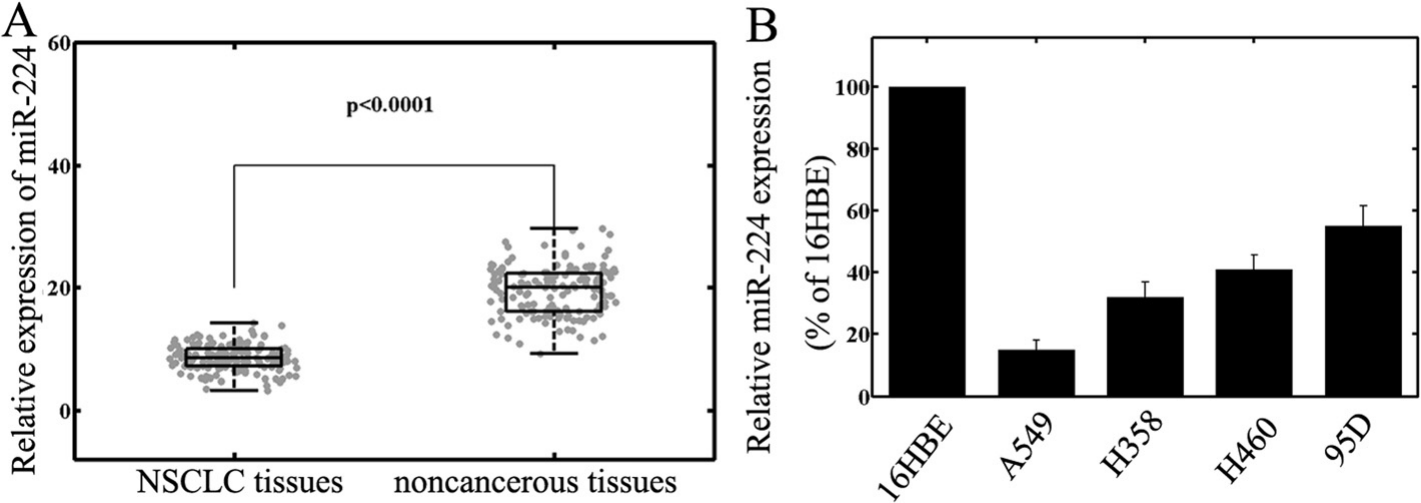
**Expression of miR-224 in non–small cell lung cancer (NSCLC) tissues and cell lines.** The expression levels were measured by qRT-PCR. **A** MiR-224 expression was significantly lower in NSCLC tissues than in the corresponding non-cancerous tissues. MiR-224 expression levels were calculated by the 2^−ΔCt^ method and normalized to U6 small nuclear RNA. **B** miR-224 expression was down-regulated in NSCLC cell lines A549, H460, 95D, and H358, compared to normal human bronchial epithelial cell line 16HBE.

The expression levels of miR-224 in primary NSCLC, corresponding adjacent normal lung tissues, human NSCLC cell lines A549, H460, 95D, and H358, and normal human bronchial epithelial cell line 16HBE were detected by qRT-PCR and normalized to U6 small nuclear RNA. As in Figure 1A, the results showed that the expression levels of miR-224 were significantly lower in NSCLC specimens (mean ± SD: 8.1 ± 2.1) than those in the corresponding adjacent non-cancerous tissues (mean ± SD: 19.5 ± 3.9; P <0.001). The miR-224 expression in four NSCLC cell lines was also clearly downregulated (Figure 1B). The A549 cell line, which possessed the lowest levels of miR-224 expression among all tested cell lines, was selected for further studies.

### miR-224 expression and clinicopathologic features in NSCLC

The associations of miR-224 expression with various clinicopathological parameters of NSCLC tissues were summarized in Table 1. Using the median miR-224 expression in all 115 NSCLC patients as a cutoff, the patients were divided into high miR-224 expression group and low miR-224 expression group. As shown in Table 1, miR-224 expression level was lower in samples with lymph node metastasis (P = 0.002) and advanced TNM stage (P <0.001). No significant difference was observed between miR-224 expression and patients’ age, gender, smoking status, cell types, T stage, and tumor differentiation.

### Down-regulation of miR-224 confers poor prognosis in patients with NSCLC

**Figure 2:**
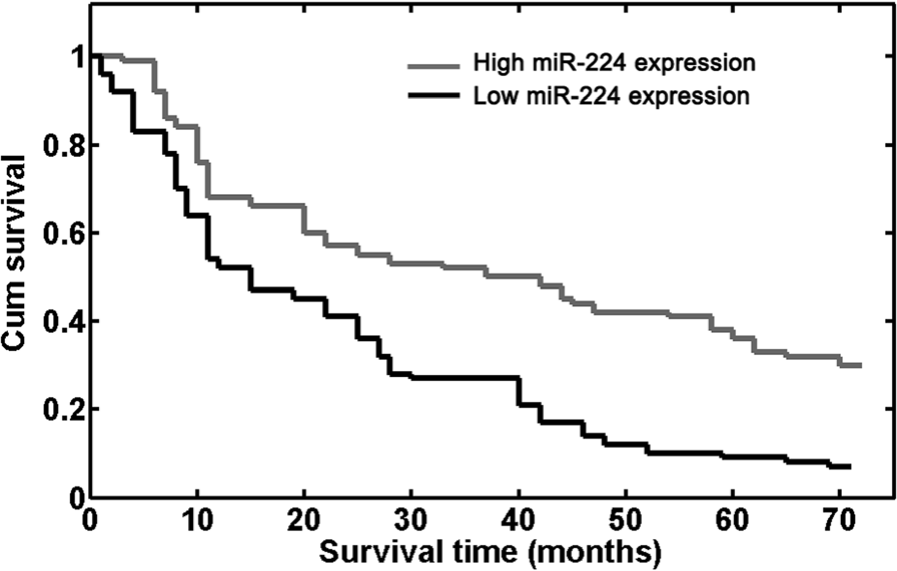
**Overall survival curves for two groups defined by low and high expression of miR-224 in patients with non–small cell lung cancer (NSCLC).** Low miR-224 expression levels were significantly associated with poor outcome (P <0.001, log-rank test).

We further evaluated whether miR-224 expression had prognostic potential for OS of NSCLC patients. Using the Kaplan–Meier method and logrank test, we found that the survival rate of patients with high miRNA-224 expression was higher than that of patients with low miRNA-224 expression (P <0.001; Figure 2). Besides, the survival benefits were also found in those with negative N classification (P = 0.022) and early TNM stage (P <0.001; Table 2).

**Table 2:** **Univariate and multivariate analysis of overall survival in 115 patients with non–small cell lung cancer**

**Variables**	**Univariate log-rank test (p)**	**Cox multivariable analysis (P)**	**Relative risk (RR)**
Age at diagnosis (years)			
<60 vs. ≥60	0.62	——	——
Gender			
Male vs. Female	0.45	——	——
Smoking status			
smoker vs never smoked	0.34	——	——
Histological type			
Squamous cell carcinoma vs Others	0.58		
Histological grade			
(G1 + G2) vs G3	0.19	——	——
T classification			
T_1+2_ vs T_3_	0.16	——	——
N classification			
Positive vs negative	0.022	0.032	5.156
TNM stage			
I-II vs III	< 0.001	0.008	9.328
MiR-224 expression			
High vs low	< 0.001	0.015	7.514

Multivariate Cox regression analysis enrolling above-mentioned significant parameters revealed that miR-224 expression (relative risk [RR] 7.514; P = 0.015), lymph node metastasis (RR 5.156; P = 0.032), and TNM stage (RR 9.328; P = 0.008) were independent prognostic markers for OS of NSCLC patients (Table 2).

### Effects of miR-224 on the proliferation, apoptosis, invasion and migration of A549 cells

**Figure 3:**
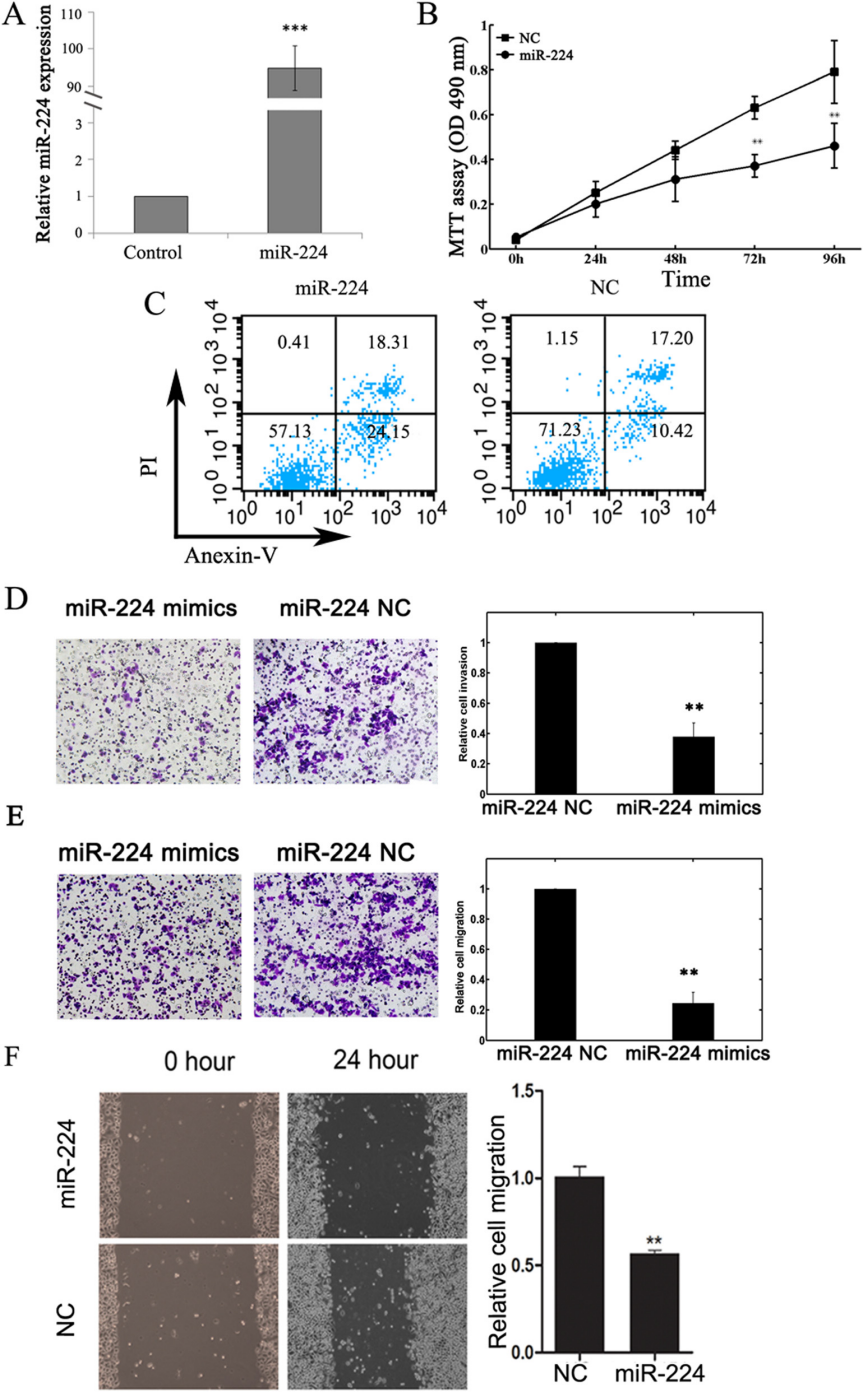
**Effects of miR-224 mimics transfection on cell proliferation, apoptosis, invasion, and migration of A549 cells. (A)** The expression level of miR-224 in miR-224 mimics transfected cells was significantly higher compared with NC transfected cells. qRT-PCR was done to detect the expression of miR-224. U6 RNA was used as an internal control. ***p <0.001. **(B)** Cell proliferation was measured by MTT assays in A549 cells transfected with miR-224 mimics or negative control. Data represent the mean ± SD of the experiments performed in triplicate. **p <0.01. **(C)** Apoptosis of A549 cells was detected by flow cytometric analysis after transfection with miR-224 mimics or negative control. **(D, E)** miR-224 suppressed A549 cell invasion and migration *in vitro*. The Matrigel invasion and migration assays showed that the number of invaded or migrated cells was significantly lower in the miR-224-transfected group than in the NC-transfected group. **p <0.01. **(F)** Scratch migration assay confirmed the inhibitory effect of miR-224 on A549 cell migration.

At last, we assessed the biological role of miR-224 in A549 cells. As shown in Figure 3A, the expression level of miR-224 in miR-224 mimics transfected cells was significantly higher compared with NC transfected cells (P <0.001). MTT assay showed that cell proliferation was significantly impaired after miR-224 mimics transfection (Figure 3B). We also observed promoted cell apoptosis in miR-224 mimics transfected cells (Figure 3C).

Transwell invasion and migration assays were performed to investigate whether miR-224 had a direct influence on A549 cell migration and invasion. As shown in Figure 3D and E, up-regulation of miR-224 impeded cell invasion/migration compared with control. Scratch migration assay also confirmed the inhibitory effect of miR-224 on A549 cell migration (Figure 3F).

## Discussion

Lung cancer is a malignant tumor that seriously threatens human health. It is of great significance to investigate molecular and cellular mechanisms of lung cancer, and to identify novel genetic or protein markers for accurate diagnosis and prediction of prognosis. In the current study, we firstly observed that miR-224 was down-regulated in NSCLC compared with adjacent noncancerous tissues. Then, decreased miR-224 expression was significantly correlated with aggressive clinicopathological features. Moreover, the Kaplan-Meier analysis revealed that NSCLC patients with low miR-224 expression tend to have shorter OS. Multivariate Cox regression analysis identified miR-224 expression level as an independent prognostic factor for OS of NSCLC patients. Finally, *in vitro* functional assays demonstrated that upregulation of miR-224 expression in A549 cells was able to reduce cell proliferation, invasion, and migration, and promote cell apoptosis. To the authors’ knowledge, this is the first report regarding the clinical significance and functional attributes of miR-224 in NSCLC.

MiR-224 has been shown its tumor-suppressor functions in several cancers. Upraity et al. reported that miR-224 was downregulated in glioblastoma tumor tissues and cell lines [19]. Upregulation of miR-224 was found to reduce clonogenic potential of glioblastoma cells and enhance radiation sensitivity. Lower miR-224 expression showed significant correlation with poorer survival. Lin et al. revealed that reduced expression of miR-224 in prostate cancer was associated with metastasis, high PSA level, high Gleason scores, and poor biochemical recurrence-free survival [25]. Forced expression of miR-224 suppressed prostate cancer cell proliferation, invasion and migration, and promoted cell apoptosis [17,25]. MiR-224 expression has also been found to correlate inversely with tumor stage and lymph node metastasis as well as survival times in colorectal cancer patients [26]. Metastatic colorectal cancer cells (SW620) transfected with miR-224 mimics had reduced migration and motility *in vitro* and formed smaller tumors with fewer metastases in mice model. Furthermore, miR-224 upregulation enhances radiation sensitivity of medulloblastoma cells [27], and a 13-gene miRNA signature including increased miR-224 levels would predict good response of lung cancer cells to EGFR inhibitor erlotinib treatment [28].

In contrast to the tumor-suppressive properties mentioned above, miR-224 also acts as an oncogene in some other cancers. Overexpression of miR-224 in human hepatocellular carcinoma was associated with promoted cell migration and invasion and poorer patient survival [20,29]. In cervical cancer, miR-224 expression was significantly higher in the cancerous tissues of patients with poor differentiation, lymph node metastasis, vascular invasion, advanced FIGO stage, and shorter overall survival [23]. In addition, the upregulation of miR-224 was also shown in breast cancer [30], clear cell renal cell carcinoma [31], pancreatic ductal adenocarcinoma [22], and bladder cancer [32]. So, miR-224 plays diverse functions in cancer pathogenesis and progression, and the role of miR-224 should be tumor specific and possibly dependent on its targets in different cancer types.

Previous research has identified many oncogenes or tumor suppressor genes as direct targets of miR-224, such as apoptosis inhibitor 5 (API5) [19], Homeobox D10 (HOXD10) [20], SMAD family member 4 (SMAD4) [33], SMAD family member 5 (SMAD5 [18], sarcolemma-associated protein (SLMAP) [18], Type 1 iodothyronine deiodinase (DIO1) [21], tumour protein D52 (TPD52) [17], tribbles homolog 1 (TRIB1) [25], chemokine (C-X-C motif) receptor 4 (CXCR4) [34], hypoxia-inducible factor 1 (HIF1A) [35], Raf kinase inhibitor protein (RKIP) [30], and cell division control protein 42 (CDC42) [36]. It is now clear that miRNAs execute their oncogenic or tumor suppressive functions by regulating the expression of target genes. However, an average miRNA can have more than 100 targets [37], and more than one miRNA can converge on a single transcript target [38]. Therefore, the potential regulatory circuitry afforded by miR-224 is enormous, and the accurate mechanisms on how miR-224 influences NSCLC progression need further clarification.

## Conclusion

In conclusion, our results revealed that miRNA-224 was down-regulated in NSCLC cell lines and clinical samples. Decreased miRNA-224 expression was associated with aggressive progression and poor prognosis. Restored miR-224 expression in A549 cells exhibited anti-tumor effects *in vitro*. These findings demonstrate that miRNA-224 could not only be useful as a novel biomarker but also serve as a potential target for gene therapy of NSCLC.
