# Expression of Concern to: Decreased microRNA-224 and its clinical significance in non-small cell lung cancer patients

**DOI:** 10.1186/s13000-019-0824-2

**Published:** 2019-05-30

**Authors:** Dan Zhu, Hui Chen, Xiguang Yang, Weisong Chen, Linying Wang, Jilin Xu, Long Yu

**Affiliations:** Department of Respiratory Medicine, Jinhua Municipal Central Hospital, Jinhua, 321000 People’s Republic of China

**Expression of Concern: Diagn Pathol (2014) 9:198**

**DOI 10.1186/s13000-014-0198-4**

The Editor-in-Chief is issuing an editorial expression of concern to alert readers that the article, Decreased microRNA-224 and its clinical significance in non-small cell lung cancer patients, Diagnostic Pathology 2014 9:198 [1] contains substantial overlap with article MiR-206 functions as a tumor suppressor and directly targets K-Ras in human oral squamous cell carcinoma Lin et al. in Onco Targets Therapy 2015; 8: 1773–1783 and Fig. 3 shows similarity to Fig. 3 in article, Decreased microRNA-452 expression and its prognostic significance in human osteosarcoma, World Journal of Surgical Oncology 2016, 14:8. As the authors have not responded to correspondence asking them for an explanation, this matter has now been referred to the authors’ institution for further investigation.
